# Urinary Proteomics Identifies ADAMDEC1 as a Non-invasive Biomarker of Unstable Carotid Atherosclerotic Plaques

**DOI:** 10.1016/j.mcpro.2026.101624

**Published:** 2026-07-17

**Authors:** Tian Yu, Qi Qin, Yumei Zhou, Mei Zhang, Qiujuan Zhang, Shilun Yang, Yinzhong Ma, Baofeng Xu, Rui Liu

**Affiliations:** 1 Center for International Medical Diagnosis and Treatment, China-Japan Union Hospital of Jilin University, Jilin University, Changchun, China; 2 Research Center for Protein and Cell-Based Drugs, Institute of Biomedicine and Biotechnology, Shenzhen Institutes of Advanced Technology, Chinese Academy of Sciences, Shenzhen, China; 3 Department of Neurosurgery, The First Affiliated Hospital of Zhengzhou University, Zhengzhou University, Zhengzhou, China; 4 Department of Stroke Center, First Hospital of Jilin University, Jilin University, Changchun, China

**Keywords:** ADAM-like decysin 1, carotid atherosclerosis, macrophage-associated inflammation, non-invasive biomarker, urinary proteomics

## Abstract

Rupture of vulnerable carotid atherosclerotic plaques (CAPs) is a major precipitating cause of ischemic stroke. Current imaging-based risk stratification is useful for anatomical and structural assessment, but it does not readily provide noninvasive access to plaque-associated molecular information. Here, we aimed to identify and validate a noninvasive urinary biomarker of CAP instability and to explore its biological relevance. We employed a multi-stage study design integrating untargeted urinary proteomics data-independent acquisition mass spectrometry in a discovery cohort of 179 patients with systematic interrogation of plaque tissue proteomic and transcriptomic datasets. Candidate proteins were further evaluated by ELISA in an independent cohort of 225 individuals, including patients with stable or unstable CAPs and healthy controls. Cellular and tissue-level analyses included single-cell RNA sequencing re-analysis, human plaque validation by immunohistochemistry, western blotting, and immunofluorescence, as well as *in vitro* macrophage phenotypic assays. Urinary proteomic profiling identified 227 shared differential proteins associated with plaque instability. In the independent validation cohort, urinary ADAM-Like Decysin 1 (ADAMDEC1) was confirmed as a candidate urinary marker, showing a stepwise increase from healthy controls to stable and unstable CAPs. Urinary ADAMDEC1 showed robust discriminatory performance for unstable plaques (AUC = 0.882, 95% CI 0.822–0.941). Higher urinary ADAMDEC1 levels were associated with symptomatic status and plaque vulnerability features, while higher intraplaque *ADAMDEC1* expression was associated with adverse cerebrovascular outcomes. Single-cell and human plaque analyses showed that *ADAMDEC1* was enriched in inflammatory macrophage-associated plaque regions, and *in vitro* assays supported an association between ADAMDEC1 and pro-inflammatory macrophage polarization. Urinary ADAMDEC1 is a noninvasive biomarker associated with carotid plaque instability. Integrated tissue, single-cell, and macrophage assays support a link between ADAMDEC1 and macrophage-associated inflammatory features in vulnerable plaques.

## Highlights

• Urinary proteomics identified ADAMDEC1 as a marker of unstable carotid plaques.
• Urinary proteomic revealed inflammatory and matrix-remodeling signatures.
• Cross-omics analyses linked urinary ADAMDEC1 to inflammatory plaque macrophages.
• Human plaque and macrophage assays supported the biological relevance of ADAMDEC1.

## Graphical Abstract

**Figure:**
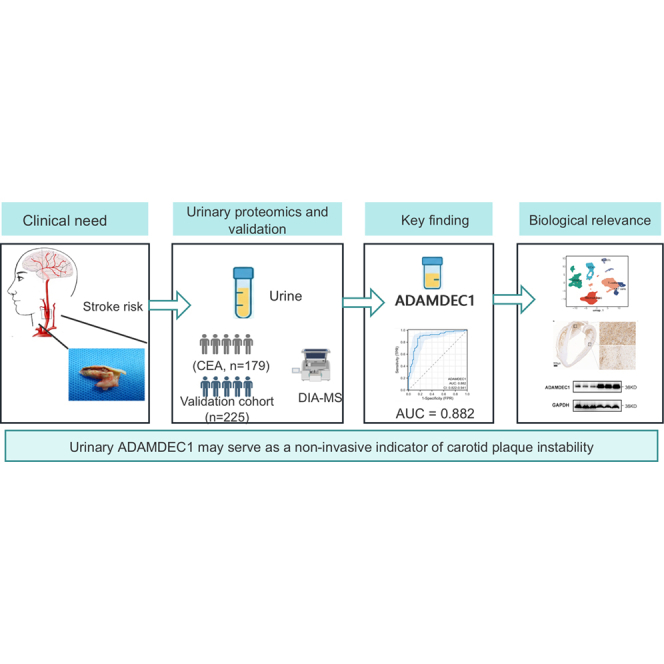

## In Brief

Using untargeted urinary proteomics and independent clinical validation, this study identified urinary ADAMDEC1 as a non-invasive biomarker associated with carotid plaque instability. Urinary proteomic profiling further revealed coordinated inflammatory and matrix-remodeling signatures associated with plaque instability. Integration with human plaque transcriptomic, proteomic, single-cell, and tissue-level analyses linked ADAMDEC1 to inflammatory macrophages, providing biological context for its translational potential in plaque risk assessment.

Carotid atherosclerotic plaque instability is a major determinant of ischemic stroke and other cerebrovascular events (1). Traditionally, the degree of luminal stenosis has been utilized in guiding clinical decision-making. Growing evidence, however, are indicating that plaque composition, inflammatory activity, and biological vulnerability—rather than stenosis severity alone—critically influence the risk of plaque rupture and downstream embolic events (1, 2, 3). Accordingly, improving the identification of unstable carotid plaques before clinical events occurrence remains a central yet unresolved challenge in vascular medical practice.

Current approaches for assessing plaque instability rely primarily on imaging modalities, including ultrasound, computed tomography angiography, and magnetic resonance imaging. These techniques provide valuable structural and morphological information but hold limitations in capturing dynamic biological processes within the plaque, such as inflammatory activation, extracellular matrix remodeling, and immune cell infiltration (4, 5, 6). Blood-based biomarkers reflecting systemic inflammation or proteolytic activity, including C-reactive protein and matrix metalloproteinases, have been explored; however, their clinical performance has been constrained by limited specificity for plaque-level pathology and susceptibility to systemic confounders (7, 8). In contrast, molecular profiling of excised carotid plaques has yielded important mechanistic insights into plaque vulnerability (9, 10), yet these approaches are inherently invasive and unsuitable for longitudinal or population-level risk assessment.

Urine represents an attractive yet underexplored source of biomarkers for vascular diseases (11). As a noninvasive biofluid, urinary proteins are highly stable and, unlike plasma, are not subject to strict homeostatic regulation, thereby facilitating the accumulation of sensitive biomarkers reflecting systemic inflammation and tissue remodeling (12, 13). Advances in mass spectrometry–based proteomics, particularly data-independent acquisition approaches, now enable deep, reproducible, and quantitative profiling of the urinary proteome (14, 15, 16). Urinary proteomics has shown promise in reflecting pathological processes in renal, cardiovascular, and inflammatory diseases (15, 17, 18); however, whether urinary proteomic profiling can capture molecular signatures that are associated with carotid plaque instability has yet to be investigated.

In the present study, we employed a multi-stage urinary proteomic strategy to identify and validate biomarkers associated with carotid plaque instability. Using data-independent acquisition mass spectrometry, we first performed unbiased discovery profiling in a well-characterized clinical cohort, followed by internal validation and confirmation in an independent patient population. We then integrated these findings with human carotid plaque transcriptomic, single-cell, and histological analyses to define their tissue and cellular context. Finally, targeted *in vitro* experiments were performed to assess potential functional relevance. Through these integrative approaches, we identified urinary ADAM-Like Decysin 1 (ADAMDEC1) as a candidate noninvasive biomarker associated with unstable CAPs. In addition, our findings provide biological context linking urinary ADAMDEC1 to macrophage-associated inflammatory features in vulnerable atherosclerotic lesions.

## Experimental Procedures

### Ethical Approval

This study was approved by the Ethics Committee of the First Hospital of Jilin University (No. 2025335) and was conducted in accordance with the Declaration of Helsinki. Written informed consent was obtained from all participants prior to enrollment.

### Experimental Design and Statistical Rationale

This study was designed as a multi-stage biomarker discovery and validation investigation. Urinary proteomic profiling by data-independent acquisition mass spectrometry was first performed in a discovery cohort of 179 patients undergoing carotid endarterectomy. To assess the reproducibility of the proteomic signal within the discovery dataset, the cohort was retrospectively divided into a training set (n = 120; 41 stable and 79 unstable plaques) and an internal validation set (n = 59; 20 stable and 39 unstable plaques). Candidate biomarkers emerging from the discovery workflow were then evaluated in an independent validation cohort of 225 individuals, including patients with stable carotid atherosclerotic plaques (CAPs), unstable CAPs, and healthy controls.

To prioritize biologically relevant candidates rather than isolated urinary signals, urinary proteomic findings were cross-referenced with published carotid plaque proteomic and transcriptomic datasets. Additional validation was performed at the transcriptomic, single-cell, and protein levels using human plaque datasets and specimens, followed by *in vitro* macrophage assays to assess phenotypic relevance.

### Study Population and Plaque Classification

Patients scheduled for carotid endarterectomy were recruited to establish the discovery cohort. Exclusion criteria were applied to minimize systemic confounding and included surgery within the preceding 6 months, active infection, malignancy, and renal or urinary tract abnormalities. First-morning midstream urine samples were collected, centrifuged to remove debris, and stored at −80 °C until analysis.

Plaque phenotype was classified based on modified American Heart Association (AHA) standards (19, 20, 21) and macroscopic morphology described in previous reports by Lovett JK *et al* and Vaisar T *et al* (22, 23). All resected plaques underwent independent pathological review by two pathologists, and discrepancies were resolved by consensus. As shown in Supplemental Fig. S1, unstable plaques were defined by the presence of surface ulceration, intraplaque hemorrhage, or a large lipid-rich necrotic core covered by a thin fibrous cap. Stable plaques were defined as smooth fibrous lesions with an intact fibrous cap and low lipid content.

### Urine Sample Preparation for Data-Independent Acquisition Mass Spectrometry

Urine samples from the discovery cohort were processed using the Urimem method described by Jia L *et al* (24). Briefly, centrifuged urine samples were passed through activated polyvinylidene difluoride membranes to adsorb urinary proteins. The protein-bound membranes were dried and vacuum-sealed until further processing. Proteins were then eluted, reduced with 20 mM dithiothreitol, alkylated with 50 mM iodoacetamide, and precipitated with cold acetone. Protein pellets were redissolved and digested overnight using the filter-aided sample preparation method with 30-kDa molecular-weight cutoff filters (Pall, Port Washington, NY) and Trypsin Gold (Mass Spectrometry Grade, Promega, Madison, WI) at an enzyme-to-protein ratio of 1:50. A pooled quality-control sample was prepared by mixing equal amounts of protein from all discovery-cohort samples and was used throughout the proteomic workflow to monitor analytical consistency.

### Data-Independent Acquisition Mass Spectrometry Acquisition

Peptide mixtures were analyzed using an Orbitrap Fusion Lumos mass spectrometer coupled to an EASY-nLC 1000 system (Thermo Fisher Scientific, Waltham, MA). To facilitate retention-time alignment across runs, indexed retention time peptides (Biognosys, Zurich, Switzerland) were added to each sample at a 1:20 ratio. The pooled quality-control sample was injected intermittently throughout the analytical sequence to assess run-to-run reproducibility.

Peptides were separated on a self-packed reversed-phase C18 capillary column (75 μm × 150 mm, 3 μm particle size) at a flow rate of 0.3 μl/min. The gradient was run from 5% to 30% buffer B over 60 min, where buffer B consisted of 0.1% formic acid in 99.9% acetonitrile. Data were acquired in DIA mode using variable isolation windows. Data were acquired in DIA mode using a variable isolation window strategy. The complete DIA isolation window scheme is provided in Supplemental Table S1. Adjacent isolation windows were designed with a 1 m/z overlap, resulting in a total cycle time of 3.15 s. Full MS scans were acquired at a resolution of 120,000 over an m/z range of 400 to 900, followed by DIA scans acquired at a resolution of 30,000. Higher-energy collisional dissociation was performed with a normalized collision energy of 32%, with an automatic gain control target of 1 × 10ˆ6 and a maximum injection time of 50 ms.

### Proteomics Data Processing and Downstream Bioinformatics

Raw DIA data were analyzed using Spectronaut Pulsar (version 12, Biognosys, Zurich, Switzerland). Peptide identification was performed by searching against the UniProt/Swiss-Prot human protein database (release 2017_09, 20,205 protein entries). Trypsin/P was specified as the digestion enzyme with a maximum of two missed cleavages.

Carbamidomethylation of cysteine was set as a fixed modification, whereas oxidation of methionine, deamidation of asparagine/glutamine, and carbamylation of lysine were specified as variable modifications. The precursor ion mass tolerance was set to 10 ppm and the fragment ion mass tolerance was set to 0.02 Da. Protein identification confidence was controlled using a Q-value cutoff of 0.01, corresponding to a protein-level false discovery rate of 1%. Only proteins identified with at least two unique peptides were retained for subsequent quantitative analyses. Complete protein identification and quantitative results, including protein accession numbers, protein descriptions, the number of unique peptides assigned to each protein, and quantitative values for all samples, are provided in Supplemental Table S2. Proteins detected in more than 50% of samples in each group were retained for downstream analysis. Missing values were imputed using the k-nearest neighbor method. Principal Component Analysis (PCA) and differential expression profiling were conducted using MetaboAnalyst 6.0 (25). Differentially expressed proteins (DEPs) were defined using a fold-change threshold >2 and an adjusted *p* value < 0.05. Hierarchical clustering was visualized using the *pheatmap* package in R. Functional enrichment analysis, including KEGG pathways and Gene Ontology terms, was carried out using the *clusterProfiler* package with *p* < 0.05. Diagnostic performance and data visualization were performed using the *pROC* and *ggplot2* packages, respectively.

### ELISA

Urinary concentrations of candidate biomarkers were quantified using commercially available ELISA kits supplied by Jiangsu Meimian Industrial Co., Ltd (China), including human ADAM-like decysin 1 (ADAMDEC1; Cat. No. MM-63977H1) and protein kinase cAMP-dependent type I regulatory subunit alpha (PRKAR1A; Cat. No. MM-63848H1). All assays were performed according to the manufacturer’s instructions. To account for urine dilution in spot urine samples, biomarker levels were normalized to urinary creatinine by calculating biomarker-to-creatinine ratios using measurements obtained from the same urine sample (26). Specifically, ELISA-derived biomarker concentrations (pg/ml) were divided by urinary creatinine concentrations (mmol/l), and the resulting values were expressed as ng/mmol creatinine.

### Systematic Mining of Tissue Transcriptomic and Proteomic Datasets

To cross-validate urinary findings with plaque tissue signatures, we integrated six published CAP proteomic datasets (10, 21, 23, 27, 28, 29) and two transcriptomic datasets (GSE43292, GSE28829) from the Gene Expression Omnibus. Overlapping targets across urinary and tissue datasets were prioritized for further evaluation. Overlapping targets were prioritized. For prognostic evaluation, Kaplan–Meier survival analysis was performed using follow-up data from 126 patients in the GSE21545 dataset (30) to assess the association between target gene expression and the incidence of cerebrovascular events. Patients were stratified into high- and low-expression groups using optimal cutpoints, and survival curves were visualized with the *ggsurvplot* function in the *survminer* R package.

### Single-Cell RNA Sequencing Analysis

We re-analyzed the scRNA-seq dataset GSE159677, comprising paired atherosclerotic core (AC) and proximal adjacent (PA) tissue specimens from patients undergoing carotid endarterectomy (31). Data preprocessing and quality control were performed using Seurat (https://satijalab.org/seurat/) (version 5.0). Cells with >10% mitochondrial transcripts or gene counts outside the range of 200–4000 were excluded. After filtering, 45,582 cells were retained (AC: 34,772; PA: 10,810) and annotated using canonical marker genes.

The macrophage compartment was then extracted (AC: 8548; PA: 1131) for re-normalization, variable-feature selection, and dimensionality reduction by PCA and uniform manifold approximation and projection. Inflammatory burden was quantified using AddModuleScore with a predefined pro-inflammatory gene set. Macrophages were further stratified according to the median level of *ADAMDEC1* expression. Visualization was performed using the ggplot2, patchwork, and ComplexHeatmap packages in R.

### Immunohistochemistry

Freshly resected CAP specimens were rinsed in cold PBS, fixed in 4% paraformaldehyde overnight, and embedded in paraffin. After deparaffinization and antigen retrieval, sections were blocked with 3% bovine serum albumin to reduce nonspecific binding. Sections were then incubated with an anti-ADAMDEC1 primary antibody (1:100; Cat. No. 17899-1-AP, Proteintech, China), followed by the corresponding secondary antibody. Immunoreactivity was visualized with 3,3′-diaminobenzidine, and nuclei were counterstained with hematoxylin. Images were acquired using a Nikon Eclipse Ci-e microscope (Nikon, Japan). Quantitative analysis was performed in Image Pro Plus using the mean optical density from three randomly selected fields per section.

### Western Blotting

Frozen CAP tissue samples (∼100 mg) were rinsed with ice-cold PBS to minimize blood contamination and homogenized in RIPA lysis buffer supplemented with protease inhibitors. Homogenates were centrifuged at 12,000*g* for 15 min at 4 °C, and the supernatants were collected for analysis. Protein concentrations were determined using the BCA assay. Equal amounts of protein were separated by SDS-PAGE and transferred to PVDF membranes. Membranes were blocked with 5% nonfat milk for 2 h at room temperature and incubated overnight at 4 °C with anti-ADAMDEC1 antibody (1:800; Cat. No. 17899-1-AP, Proteintech, China). GAPDH (1:3000; Cat. No. 60004-1-Ig, Proteintech) was used as the loading control.

### Immunofluorescence Staining

Fresh CAP specimens were fixed in 4% paraformaldehyde overnight at 4 °C, cryoprotected sequentially in 15% and 30% sucrose, embedded in OCT compound, and frozen. For cell-based assays, cultured cells were fixed with 4% paraformaldehyde for 30 min at 4 °C. After three washes in PBS, samples were blocked with 5% goat serum for 1 h at room temperature and incubated overnight at 4 °C with primary antibodies against ADAMDEC1 (1:100; Cat. No. 17899-1-AP, Proteintech), CD68 (1:100; Cat. No. 66231-2-Ig, Proteintech), and CD86 (1:100; Cat. No. A24386 PM, ABclonal). Samples were then incubated with fluorophore-conjugated secondary antibodies for 60 min at room temperature in the dark. Nuclei were counterstained with 4′,6-diamidino-2-phenylindole (Solarbio), and images were captured using a Zeiss Axio Imager Z2 microscope with Apotome.2 (Zeiss, Germany).

### Cell Culture and Macrophage Phenotypic Assays

THP-1 monocytes were obtained from Zhong Qiao Xin Zhou Biotechnology (Wuhan, China) and cultured in RPMI-1640 medium supplemented with 10% fetal bovine serum and penicillin–streptomycin at 37 °C in a humidified 5% CO2 atmosphere. Differentiation into macrophage-like cells was induced by treatment with 100 nM phorbol 12-myristate 13-acetate for 24 h. All cell cultures were routinely confirmed to be free of *mycoplasma* contamination.

For gene silencing experiments, THP-1–derived macrophages were transfected with 20 nM ADAMDEC1-targeting siRNA or negative-control siRNA using Lipofectamine RNAiMAX (Invitrogen) according to the manufacturer’s protocol. The targeting sequences synthesized by GenePharma (Shanghai, China) were as follows: si-1, 5′-GUUCCAUUCAACACCAAGUTT-3′; si-2, 5′-GCCCUAACGUGUAAACUGATT-3′. For functional stimulation assays, cells were treated with recombinant ADAMDEC1 (200 ng/ml; MedChemExpress) or lipopolysaccharide (LPS) (1000 ng/ml; Sigma-Aldrich) in complete medium.

### Flow Cytometry

THP-1–derived macrophages were treated with recombinant ADAMDEC1 (200 ng/ml) for 48 h, or transfected with control or ADAMDEC1-targeting siRNA for 24 h followed by LPS stimulation (1000 ng/ml) for 48 h. Cells were then harvested and resuspended in staining buffer consisting of PBS with 3% fetal bovine serum. Fc receptors were blocked with anti-CD16/32 antibody (BioLegend) for 10 min before staining. Cells were incubated for 45 min with fluorophore-conjugated antibodies against CD86 (BV605; Cat. No. 374202, BioLegend) and CD206 (AF488; Cat. No. 321102, BioLegend). Data were acquired using a CytoFLEX S flow cytometer (Beckman Coulter) and analyzed with FlowJo software (https://www.flowjo.com/) (version 7.6.5).

### Statistical Analysis

Data are presented as mean ± SD, median (interquartile range), or number (%), as appropriate. Between-group comparisons were performed using Student’s *t* test or the Mann–Whitney U test for 2-group analyses, and one-way ANOVA or the Kruskal–Wallis test with Tukey or Dunn post hoc correction for comparisons involving more than two groups, depending on data distribution. Categorical variables were compared using the chi-square test or Fisher’s exact test. Correlations were assessed using Pearson or Spearman coefficients as appropriate. Logistic regression and receiver operating characteristic curve analyses were used to evaluate diagnostic performance. All analyses were conducted in R (version 4.4.0), and all tests were 2-sided with *p* < 0.05 considered statistically significant.

## Results

### Urinary Proteomic Profiling Identifies Signatures Associated with Carotid Plaque Instability

**Fig. 1:**
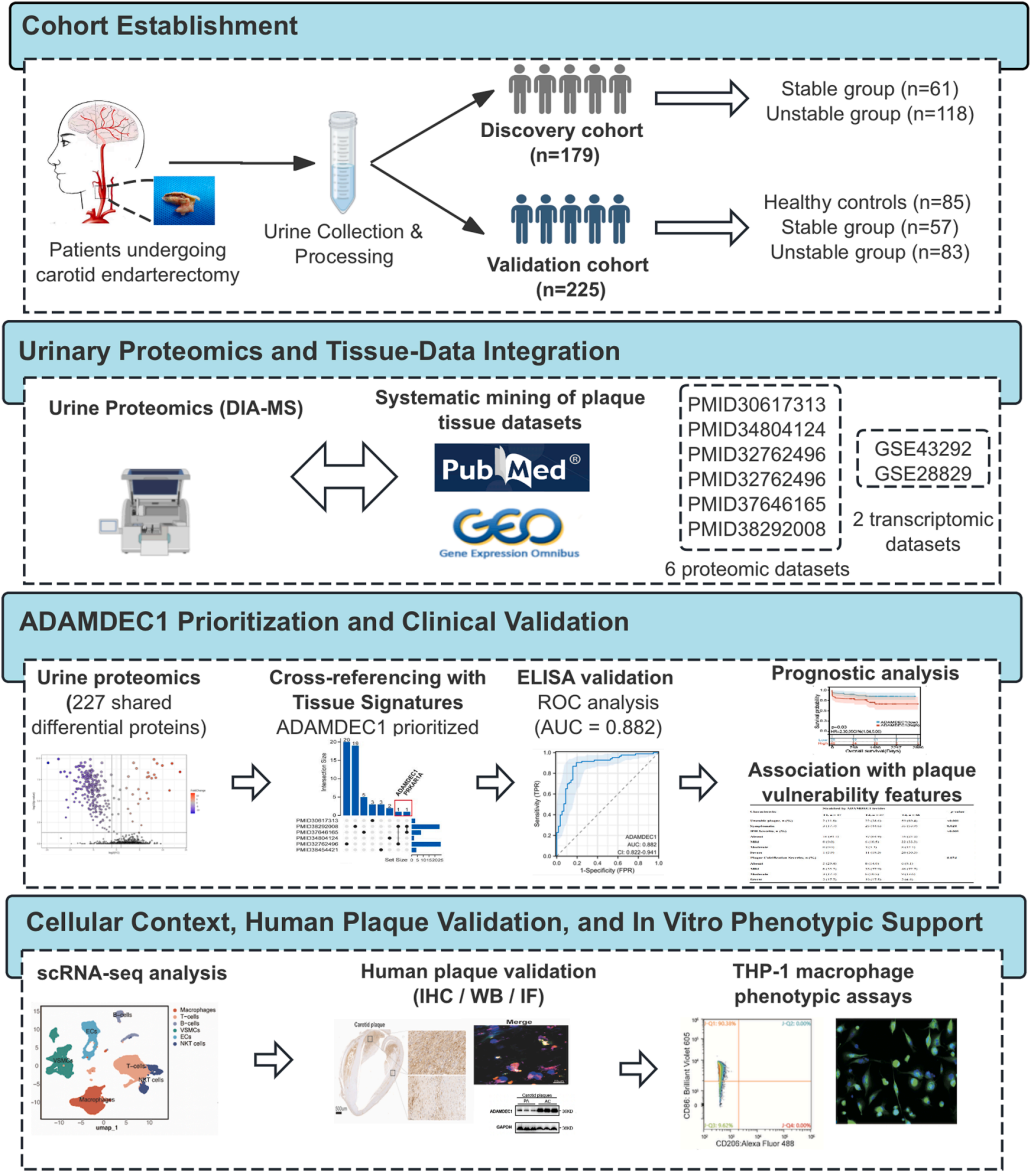
**Schematic overview of the study design.** The study comprised four phases: cohort establishment, urinary proteomics and tissue-data integration, ADAMDEC1 prioritization and clinical validation, and cellular/experimental support. A discovery cohort (n = 179) and an independent validation cohort (n = 225) were established. Urinary proteomic profiling by data-independent acquisition mass spectrometry was integrated with published plaque tissue proteomic and transcriptomic datasets. ADAMDEC1 was prioritized through cross-referencing of urinary and tissue signatures and was subsequently evaluated by ELISA, receiver operating characteristic analysis, multivariable logistic regression, prognostic analysis, and assessment of associations with plaque vulnerability features. single-cell RNA sequencing, human plaque validation (immunohistochemistry / western blotting /IF), and THP-1 macrophage assays were used to define the cellular context and provide experimental support.IF, immunofluorescence.

To systematically identify and validate noninvasive urinary biomarkers for CAP instability, we employed a multi-stage study design (Fig. 1). Urinary proteomic profiling was performed in a discovery cohort of 179 patients with CAPs, (Fig. 1 and Table 1). Baseline demographics and cardiovascular risk factors were comparable between stable and unstable groups (Table 1).

**Table 1:** Baseline demographic and clinical characteristics of the discovery cohort stratified by plaque stability

Characteristic	Training set		*p* value	Internal validation set		*p* value
	Stable (n = 41) unstable (n = 79)			Stable (n = 20) unstable (n = 39)		
Age(years)	63 (55, 72)	66 (62, 70)	0.185	66 (62, 70)	66 (62, 70)	0.690
Sex (male, n, %)	28 (68.3)	66 (83.5)	0.054	16 (80)	31 (79.5)	1.000
Body Mass Index (kg/m^2^)	22.86 (20.9, 25.0)	22.86 (21.2, 25.1)	0.419	22.67 (21.7, 25.1)	24.29 (23.2, 26.1)	0.080
Smoking, n (%)	15 (36.6)	43 (54.4)	0.070	10 (50.0)	23 (59.0)	0.389
Drinking, n (%)	11 (26.8)	35 (44.3)	0.057	8 (40)	19 (48.7)	0.432
Hypertension, n (%)	23 (56.1)	44 (55.7)	0.792	10 (50.0)	20 (51.3)	0.912
Diabetes, n (%)	7 (17.1)	18 (22.8)	0.524	11 (55.0)	12 (30.8)	0.075
Coronary heart disease, n (%)	5 (12.2)	13 (16.5)	0.568	4 (20.0)	6 (15.4)	1.000
History of stroke, n (%)	8 (19.5)	22 (27.8)	0.367	8 (40.0)	19 (48.7)	0.469
Hyperlipidemia, n (%)	3 (7.3)	7 (8.9)	1.000	4 (20.0)	4 (10.3)	0.059
Triglycerides (mmol/L)	1.28 (0.8, 1.5)	1.23 (0.9, 1.7)	0.653	1.39 (0.9, 1.6)	1.04 (0.8, 1.6)	0.476
High-Density Lipoprotein (mmol/L)	0.96 (0.8, 1.1)	0.98 (0.8, 1.1)	0.397	0.88 (0.7, 1.1)	0.96 (0.8, 1.1)	0.506
Low-Density Lipoprotein (mmol/L)	2.07 (1.7, 2.5)	2.09 (1.8, 2.4)	0.902	2.02 (1.6, 2.5)	2.05 (1.8, 2.4)	0.737
Total Cholesterol (mmol/L)	3.62 (3.1, 4.3)	3.28 (3.1, 4.1)	0.430	3.51 (3.1, 4.2)	3.51 (3.1, 3.9)	0.713

Note: Data are presented as median (IQR) for continuous variables and n (%) for categorical variables. *p* values were calculated using the Mann–Whitney *U* test for continuous variables and the Chi-square test or Fisher's exact test for categorical variables.

**Fig. 2:**
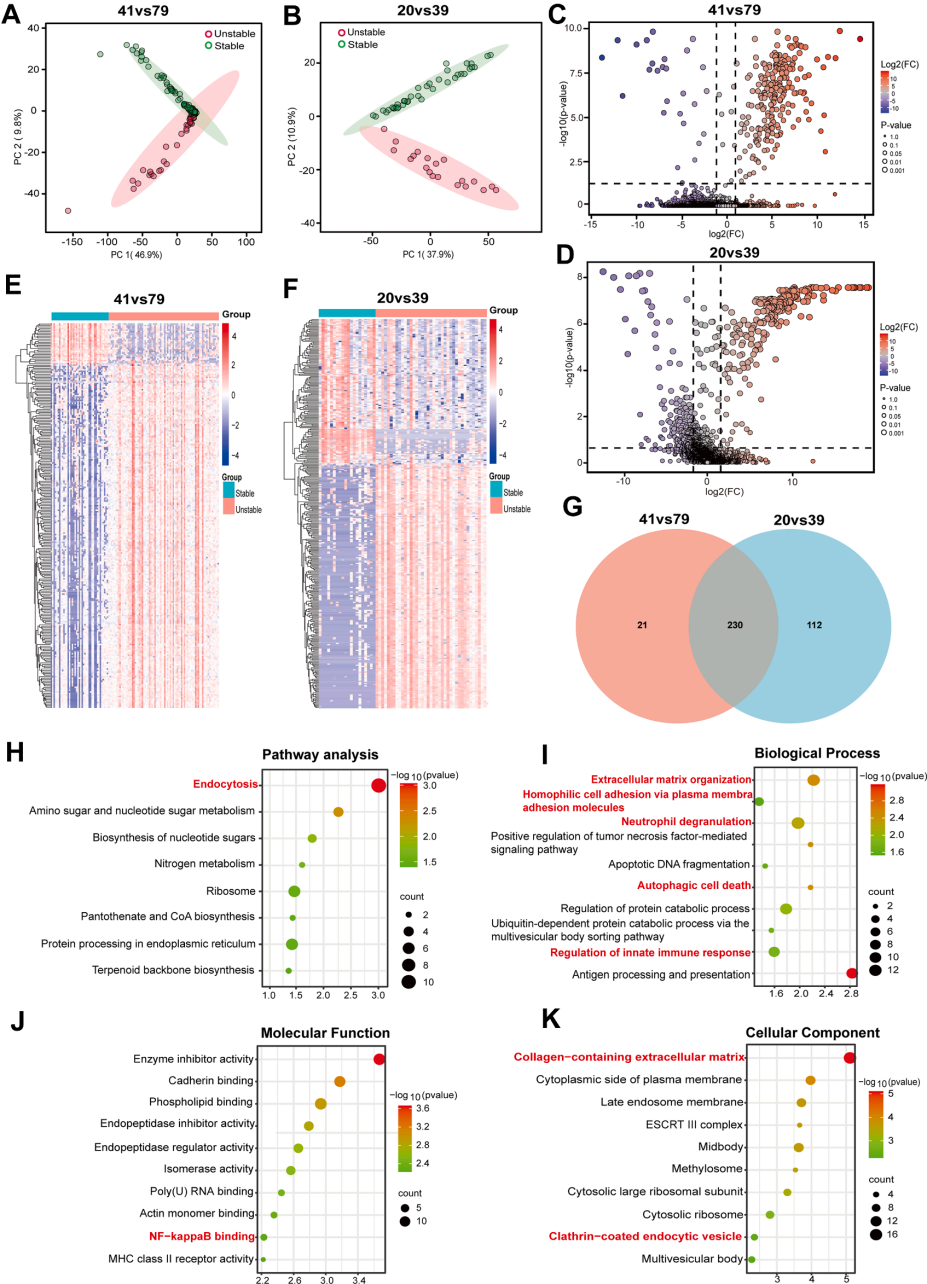
**Urinary proteomic profiling identifies reproducible molecular signatures associated with carotid plaque instability.***A* and *B,* Principal component analysis of urinary proteomic profiles showing clear separation between stable and unstable carotid atherosclerotic plaques (CAPs) in the training set (*A*) and internal validation set (*B*). *C* and *D, volcano plots* showing DEPs between stable and unstable CAPs in the training set (*C*) and internal validation set (*D*). Upregulated and downregulated proteins are highlighted in red and blue, respectively. *E* and *F,* heatmaps of DEPs showing robust stratification of stable and unstable samples in the training set (*E*) and internal validation set (*F*). *G, venn diagram* showing the overlap of DEPs between the training and internal validation sets. A total of 230 shared DEPs were identified, of which 227 showed consistent directionality across both sets and were retained as high-confidence candidates. *H,* KEGG pathway enrichment analysis of the 227 shared DEPs. Enriched pathways included extracellular matrix-related processes, inflammatory and innate immune pathways, antigen processing and presentation, endocytosis-related pathways, and intracellular protein processing programs. *I,* GO biological process enrichment analysis showing over-representation of processes related to extracellular matrix organization, regulation of innate immune responses, neutrophil degranulation, cellular protein catabolic processes, and cell adhesion. *J,* GO molecular function enrichment analysis shows enrichment of antigen binding, serine-type peptidase activity, metal ion binding, and protein-containing complex binding. *K,* GO cellular component enrichment analysis showing enrichment of collagen-containing extracellular matrix, extracellular matrix structural constituents, clathrin-coated endocytic vesicles, and major histocompatibility complex protein complexes. DEPs, differentially expressed proteins; GO, Gene Ontology.

Unsupervised multivariate analyses of data-independent acquisition mass spectrometry data demonstrated consistent separation of stable versus unstable CAPs across both subsets. PCA showed distinct clustering between the two phenotypes in the training set and the internal validation set (Fig. 2, *A* and *B*). Differential expression analysis identified 251 DEPs in the training set and 342 DEPs in the internal validation set, visualized by volcano plots (Fig. 2, *C* and *D*, Supplemental Table S3). Heatmap visualization further indicated that these DEPs robustly stratified stable versus unstable samples within each subset (Fig. 2, *E* and *F*). To prioritize reproducible signals, we performed overlap analysis between the two subsets. A total of 230 DEPs were common to both sets (Figs. 2*G*), and 227 were retained as high-confidence candidates by requiring consistent directionality of change across training and internal validation (Supplemental Table S3).

Functional enrichment analysis of the shared DEPs revealed significant over-representation of multiple biological processes relevant to plaque destabilization, including extracellular matrix organization, inflammatory and innate immune responses, antigen processing and presentation, neutrophil degranulation, endocytosis-related pathways, and intracellular protein processing catabolic programs (Fig. 2, *H–K*). Collectively, these analyses establish that urinary proteomic profiles capture disease-relevant molecular signatures associated with CAP instability, providing a robust set of reproducible candidates for subsequent multi-omics prioritization and independent clinical validation.

### Cross-Omics Prioritization and Independent Validation Identify ADAMDEC1 as a Urinary Biomarker of Unstable Carotid Plaques

**Fig. 3:**
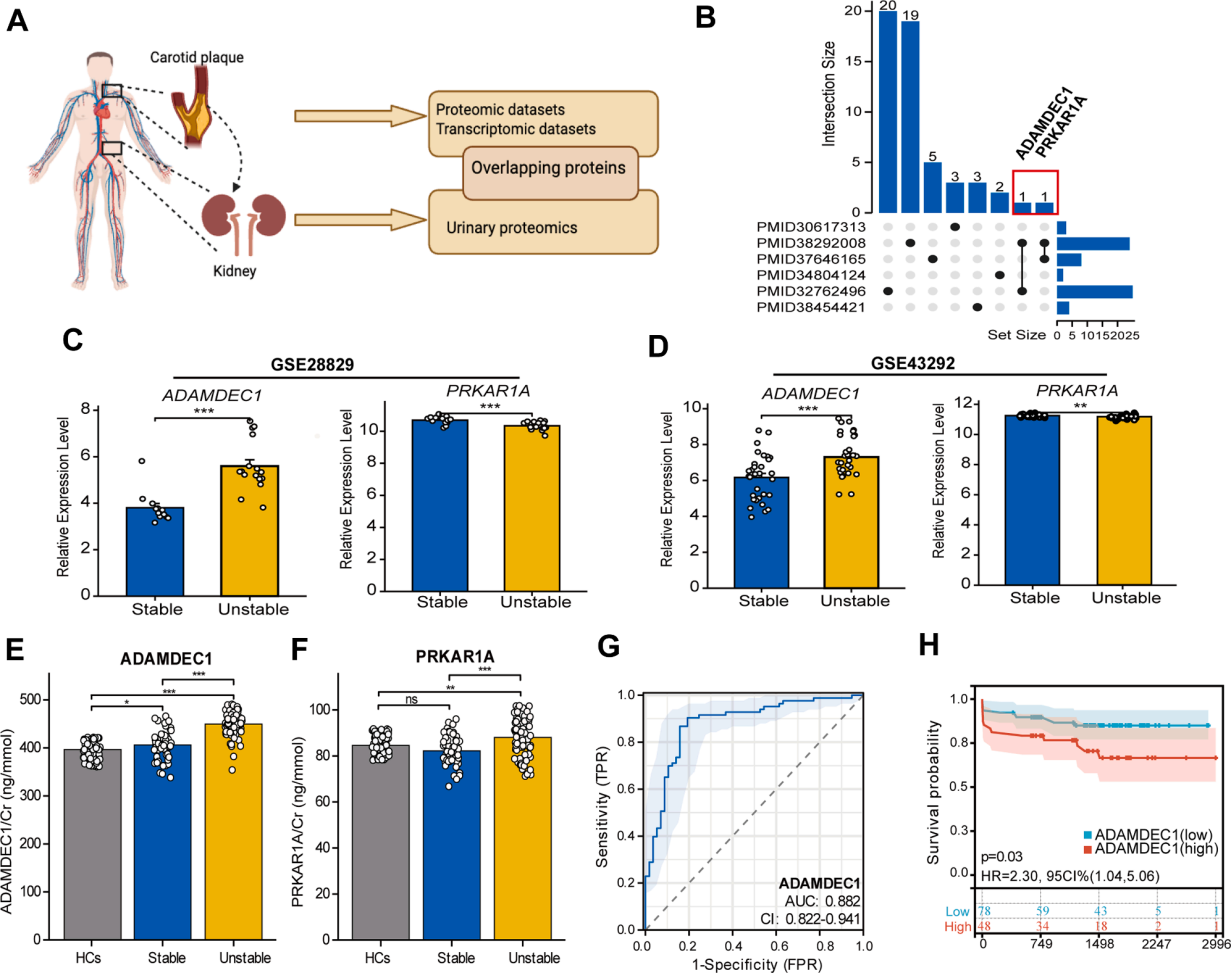
**Cross-omics prioritization and independent validation identify ADAMDEC1 as a urinary biomarker of unstable carotid plaques.***A,* schematic workflow outlining the strategy for integrating tissue-based proteomic and transcriptomic datasets with urinary proteomics to identify overlapping targets. *B,* UpSet plot revealing the intersection of DEPs across six independent published proteomic studies. The *red box* highlights ADAMDEC1 and PRKAR1A as recurrent candidate proteins identified in multiple plaque datasets. *C* and *D,* transcriptomic validation of *ADAMDEC1* and *PRKAR1A* expression in unstable *versus* stable plaques using two external Gene Expression Omnibus datasets (GSE28829 and GSE43292). *ADAMDEC1* was upregulated, whereas *PRKAR1A* was downregulated in unstable plaques. *E* and *F,* ELISA-based quantification of urinary ADAMDEC1 and PRKAR1A in the independent external validation cohort (n = 225; 85 HCs, 57 stable CAPs, and 83 unstable CAPs). Urinary ADAMDEC1 levels showed a stepwise increase from HCs to stable and unstable CAPs, whereas PRKAR1A was higher in unstable CAPs than in stable CAPs but did not differ significantly between HCs and stable CAPs. *G,* receiver operating characteristic curve showing the diagnostic performance of urinary ADAMDEC1 for discriminating unstable from stable carotid plaques. *H*, Kaplan-Meier analysis of cerebrovascular event-free survival based on intraplaque *ADAMDEC1* expression in the GSE21545 dataset (n = 126), showing that high *ADAMDEC1* expression was associated with poorer prognosis. Data in *(C* and *F)* are presented as mean ± SEM. Statistical significance was determined using Student’s *t* test for *(C* and *D)* and the Kruskal–Wallis test followed by Dunn’s post hoc test for *(E* and *F)*. ns *p* > 0.05, ∗*p* < 0.05, ∗∗*p* < 0.01, ∗∗∗*p* < 0.001. AUC, area under the curve; CAP, carotid atherosclerotic plaque; DEP, differentially expressed protein; HC, healthy control.

To prioritize urinary biomarkers with strong biological plausibility, we identified proteins consistently dysregulated in both urine and atherosclerotic plaques. As shown in Figure 3*A*, we cross-referenced our urinary DEPs with CAP proteomic data from published studies and transcriptomic profiles from Gene Expression Omnibus. Despite heterogeneity in patient definitions, analytical methods, and significance thresholds across sources (Supplemental Table S4), we retained only proteins showing concordant up- or down-regulation in unstable versus stable plaques, ensuring robustness in the overlap analysis. Analysis revealed an overlap of 55 proteins between the urinary and plaque datasets (Fig. 3*B*). Two candidates—ADAMDEC1 and PRKAR1A—were distinguished by their recurrence in multiple independent plaque datasets (Fig. 3*B*, red box). These findings were further examined at the mRNA level in two external datasets (GSE28829 and GSE43292), where *ADAMDEC1* was consistently upregulated in unstable plaques, whereas *PRKAR1A* was downregulated (Fig. 3, *C* and *D*).

To evaluate the reproducibility and discriminatory performance of the selected candidate urinary biomarkers emerging from the discovery proteomic analysis, these proteins were examined in an expanded, independent validation cohort comprising 140 patients with CAPs (57 stable vs. 83 unstable) and 85 healthy controls. Baseline demographic and clinical characteristics were broadly comparable between groups (Supplemental Table S5). Consistent with our discovery-stage findings, urinary ADAMDEC1 was significantly elevated in patients with unstable plaques compared with those with stable plaques in the validation cohort (Fig. 3*E*). Notably, urinary ADAMDEC1 showed a stepwise increase from healthy controls to stable and unstable CAPs. While urinary PRKAR1A was significantly higher in the unstable group than in the stable group, no significant difference was observed between healthy controls and patients with stable CAPs (Fig. 3*F*). Given this limited discriminatory value at the early disease stage, PRKAR1A was not included in subsequent diagnostic analyses.

Individually, urinary ADAMDEC1 effectively discriminated unstable from stable plaques, with an AUC of 0.882 (95% CI 0.822–0.941) (Fig. 3*G*). This association remained significant after progressive adjustment for confounders—including age, sex, BMI, and cardiovascular comorbidities—supporting the robustness of urinary ADAMDEC1 as an indicator of plaque instability in the validation cohort (Supplemental Table S6). To evaluate the prognostic relevance of ADAMDEC1, we analyzed follow-up data from 126 patients with CAPs using a public plaque tissue transcriptomic dataset. Higher intraplaque *ADAMDEC1* expression was significantly associated with an increased risk of cerebrovascular events (Fig. 3*H*), supporting its potential prognostic relevance. Together, these findings support the diagnostic utility of urinary ADAMDEC1 for identifying unstable carotid plaques across independent cohorts, while higher intraplaque *ADAMDEC1* expression additionally suggests potential prognostic relevance.

### Urinary ADAMDEC1 Levels are Associated with Plaque Vulnerability and Clinical Phenotypes

To further characterize the clinical relevance of urinary ADAMDEC1, we examined its associations with established indicators of carotid plaque vulnerability and patient phenotypes. Patients were stratified according to urinary ADAMDEC1 tertiles, and associations were examined across multiple clinical variables and plaque vulnerability features (Table 2). Furthermore, higher urinary ADAMDEC1 levels were associated with symptomatic status and with greater intraplaque haemorrhage (IPH) severity, as reflected by a progressive decrease in the proportion of IPH-absent plaques and an increase in severe IPH across tertiles. An association with plaque calcification was also observed, with severe calcification being less frequent and mild calcification being more common in the highest ADAMDEC1 tertile.

**Table 2:** Association of urinary ADAMDEC1 level with plaque characteristics in the validation cohort

Characteristic	Stratified by ADAMDEC1 tertiles			*p* value
	T1, n = 17	T2, n = 57	T3, n = 66	
Unstable plaque, n (%)	2 (11.8)	22 (38.6)	59 (89.4)	<0.001
Symptomatic	3 (17.7)	25 (44.6)	35 (53.9)	0.028
IPH Severity, n (%)				<0.001
Absent	16 (94.1)	37 (64.9)	16 (24.2)	
Mild	0 (0.0)	6 (10.5)	22 (33.3)	
Moderate	0 (0.0)	3 (5.3)	8 (12.1)	
Severe	1 (5.9)	11 (19.3)	20 (30.3)	
Plaque Calcification Severity, n (%)				0.024
Absent	5 (29.4)	8 (14.0)	6 (9.1)	
Mild	6 (35.3)	33 (57.9)	48 (72.7)	
Moderate	3 (17.7)	6 (10.5)	9 (13.6)	
Severe	3 (17.7)	10 (17.5)	3 (4.6)	

Note: Data are presented as n (%). *p* values were calculated using the Chi-square test or Fisher's exact test.

Definitions: IPH/Calcification Severity: Absent, no haemorrhage or calcification; Mild, volume less than one quarter; Moderate, volume between one quarter and one half; Severe, volume greater than one half.

Abbreviations: IPH, intraplaque haemorrhage; T1, tertile 1 (low); T2, tertile 2 (medium); T3, tertile 3 (high).

We next evaluated the relationships between urinary ADAMDEC1 levels and conventional cardiovascular risk factors. Urinary ADAMDEC1 levels were not significantly associated with most conventional cardiovascular risk factors, including age, sex, body mass index, smoking, hypertension, diabetes, or plasma lipid profiles (Supplemental Table S7). Together with the multivariable analyses presented above, in which the association between urinary ADAMDEC1 and plaque instability remained significant after adjustment for these covariates (Supplemental Table S6), these data suggest that the observed associations with plaque instability were not fully explained by traditional risk factors. Collectively, these analyses indicate that elevated urinary ADAMDEC1 levels are closely associated with carotid plaque vulnerability and relevant clinical phenotypes, supporting its relevance as a marker of plaque instability.

### *ADAMDEC*1 is Enriched in Inflammatory Macrophages within Advanced Carotid Plaques

**Fig. 4:**
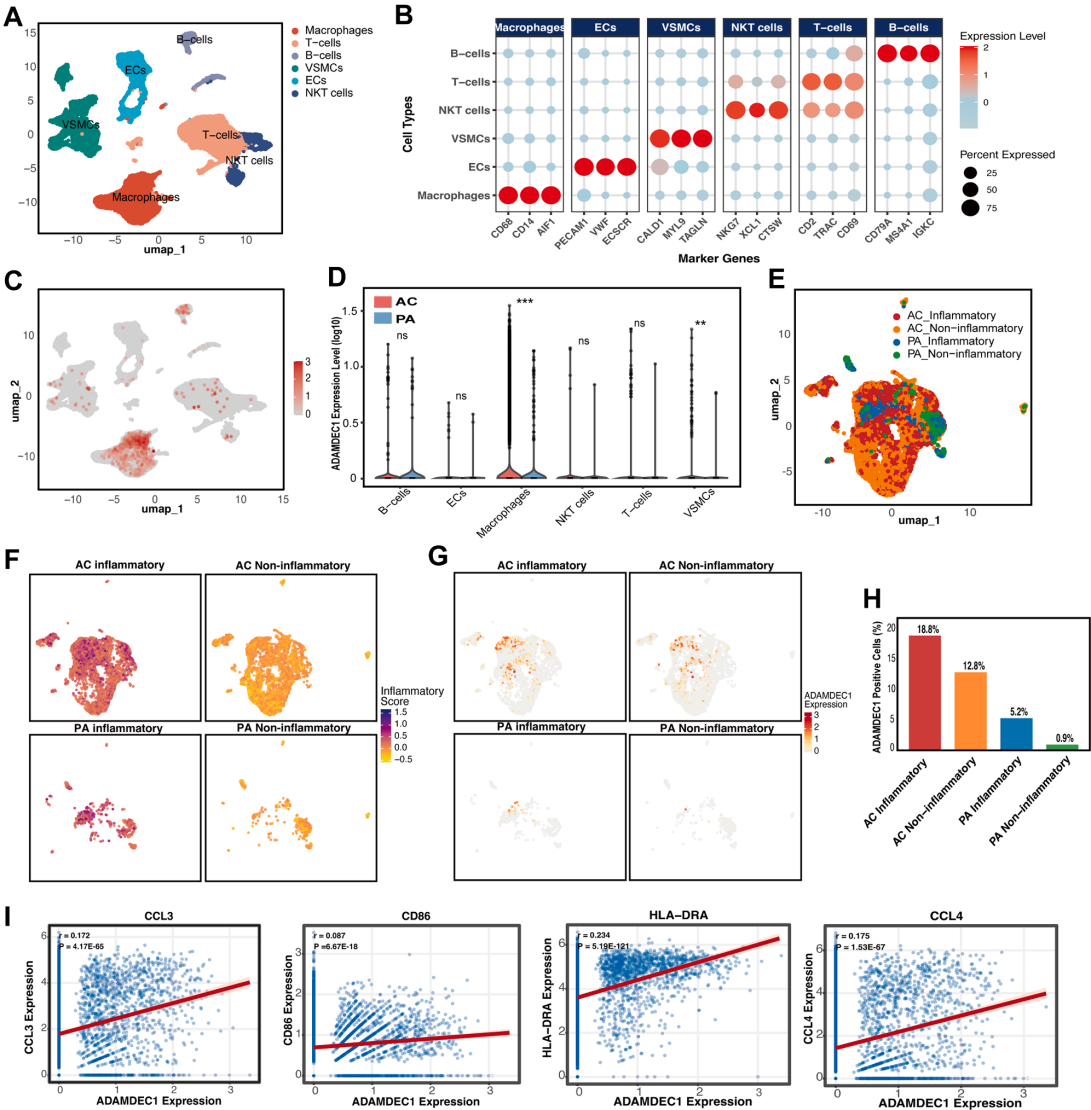
***ADAMDEC1* is enriched in inflammatory macrophages within advanced carotid plaques.***A,* UMAP visualization of the single-cell transcriptomic landscape in human carotid atherosclerotic plaques, identifying six major cell lineages. *B, Dot plot* showing the expression intensity and proportion of canonical marker genes used to annotate major cell populations. *C, Feature plot* showing that *ADAMDEC1* expression is predominantly restricted to the macrophage cluster. *D, Violin plots* comparing *ADAMDEC1* expression across major cell populations between AC and PA tissues, showing significantly higher expression in macrophages from AC tissues. *E,* Uniform Manifold Approximation and Projection projection of the macrophage subpopulation, stratified into AC inflammatory, AC noninflammatory, PA inflammatory, and PA noninflammatory subsets. *F,* distribution of inflammatory signature scores across macrophage subsets projected onto the Uniform Manifold Approximation and Projection embedding. *G, feature plots* showing preferential enrichment of *ADAMDEC1* expression in inflammatory macrophage subsets, particularly in AC inflammatory macrophages. *H*, quantification of the proportion of *ADAMDEC1*-positive cells across macrophage subsets, showing the highest proportion in AC inflammatory macrophages. *I, Scatter plots* showing positive correlations between *ADAMDEC1* expression and the pro-inflammatory genes *CCL3*, *CD86*, *HLA-DRA*, and *CCL4* in plaque macrophages. Pearson’s correlation coefficients (r) and *p* values are indicated. Data in (*D*) are presented as mean ± SEM. Statistical significance was determined using the Wilcoxon rank-sum test for (*D*). ns *p* > 0.05, ∗*p* < 0.05, ∗∗*p* < 0.01, ∗∗∗*p* < 0.001. AC, atherosclerotic core; PA, proximal adjacent tissue.

To define the cellular distribution and tissue context associated with urinary ADAMDEC1, we re-analyzed plaque single-cell RNA-seq data from patients with AC and PA tissues. Unsupervised clustering identified major plaque-associated populations, including macrophages, T cells, B cells, VSMCs, ECs, and NKT cells (Fig. 4, *A* and *B*). Across all cell types, *ADAMDEC1* expression was highly restricted to the macrophage compartment (Fig. 4*C*) and was significantly higher in macrophages derived from AC than from PA tissues (Fig. 4*D*).

Given the heterogeneity of plaque macrophages, we next subclustered macrophages into inflammatory and noninflammatory subsets across AC and PA tissues (Fig. 4*E*). Consistent with this classification, inflammatory signature scores were markedly increased in inflammatory macrophage subsets (Fig. 4*F*), and *ADAMDEC1* expression was preferentially enriched in these subsets, particularly in AC inflammatory macrophages (Fig. 4*G*). Quantification further showed that *ADAMDEC1*-positive cells were most frequent in the AC inflammatory macrophage subset, followed by AC noninflammatory, PA inflammatory, and PA noninflammatory subsets (Fig. 4*H*). At the transcriptional level, *ADAMDEC1* expression correlated positively with canonical pro-inflammatory genes, including *CCL3*, *CD86*, *HLA-DRA*, and *CCL4*, within plaque macrophages (Fig. 4*I*), supporting a close association between *ADAMDEC1* and inflammatory macrophage programs in human carotid atherosclerotic lesions.

### Human Plaque Validation and *in vitro* Assays Support a Link Between ADAMDEC1 and Pro-inflammatory Macrophage Activation

**Fig. 5:**
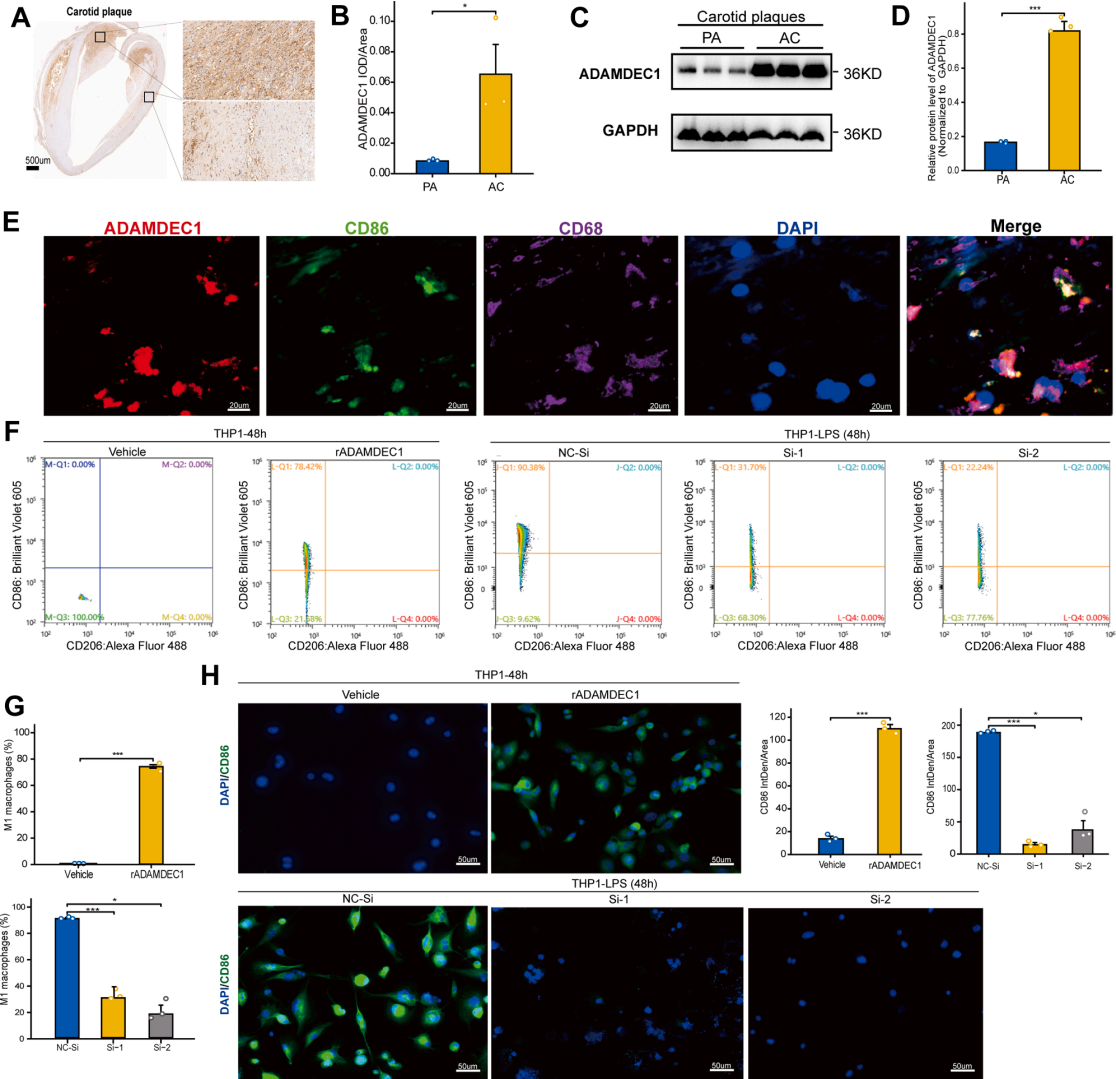
**Human plaque validation and *in vitro* assays support an association between ADAMDEC1 and pro-inflammatory macrophage polarization.***A,* representative immunohistochemical staining of ADAMDEC1 in human carotid plaque specimens, showing stronger staining in AC than in PA regions. The scale bar represents 500 μm in the overview image and 50 μm in the enlarged panels. *B,* quantification of ADAMDEC1 immunoreactivity by integrated optical density /area, confirming increased staining intensity in AC compared with PA tissues. *C,* representative Western blot images showing higher ADAMDEC1 protein abundance in AC than in PA tissues. GAPDH was used as the loading control. *D,* quantification of western blotting results, showing increased relative ADAMDEC1 protein levels in AC tissues. *E,* IF staining showing colocalization of ADAMDEC1 with CD68-and CD86-positive macrophages in human carotid plaques. The scale bar represents 20 μm. *F,* representative flow cytometry plots showing macrophage polarization in THP-1 derived macrophages following rADAMDEC1 treatment or ADAMDEC1 knockdown under lipopolysaccharide stimulation. *G,* quantification of the proportion of M1-like macrophages, showing increased polarization following rADAMDEC1 treatment and reduced polarization following ADAMDEC1 silencing. *H,* representative IF images of CD86 expression in THP-1-derived macrophages after rADAMDEC1 treatment or ADAMDEC1 knockdown. The cale bar represents 50 μm. *I,* quantification of CD86 fluorescence intensity, showing enhanced CD86 expression after rADAMDEC1 treatment and reduced expression after ADAMDEC1 knockdown. Data in (*B*, *D*, *G*, *I*) are presented as mean ± SEM. Statistical significance was determined using Student’s *t* test for two-group comparisons (*B*, *D*, G *top*, *I top*) and one-way ANOVA followed by Tukey’s post hoc test for multi-group comparisons (*G bottom, I bottom*). ns *p* > 0.05, ∗*p* < 0.05, ∗∗*p* < 0.01, ∗∗∗*p* < 0.001. AC, atherosclerotic core; IF, immunofluorescence; PA, proximal adjacent tissue; rADAMDEC1, recombinant ADAMDEC1.

We next validated these observations at the protein level in human carotid plaque specimens. Immunohistochemistry showed stronger ADAMDEC1 staining in AC than in PA regions (Fig. 5*A*), which was confirmed by quantitative integrated optical density analysis (Fig. 5*B*). Consistently, western blotting further supported increased ADAMDEC1 protein abundance in AC compared with PA tissues (Fig. 5, *C* and *D*). Immunofluorescence (IF) staining demonstrated colocalization of ADAMDEC1 with CD68-and CD86-positive macrophages (Fig. 5*E*). Together, these results indicate that ADAMDEC1 is enriched in macrophage-rich inflammatory regions of advanced human carotid plaques.

To investigate the functional relevance of ADAMDEC1 in macrophage polarization, we next modulated ADAMDEC1 levels in THP-1-derived macrophages. Flow cytometric analysis demonstrated that treatment with recombinant ADAMDEC1(rADAMDEC1) significantly increased the proportion of pro-inflammatory M1-like macrophages, whereas ADAMDEC1 silencing attenuated LPS-induced M1 polarization (Fig. 5, *F* and *G*). These gain- and loss-of-function effects were further corroborated by IF staining, which showed enhanced CD86 expression following rADAMDEC1 treatment and reduced CD86 signal upon ADAMDEC1 knockdown (Fig. 5, *H* and *I*). Together, these findings support an association between ADAMDEC1 and pro-inflammatory macrophage polarization in THP-1-derived macrophages.

## Discussion

In this study, we performed a comprehensive urinary proteomic analysis to identify a noninvasive biomarker associated with carotid plaque instability. Through a multi-stage discovery and validation strategy, we found that urinary ADAMDEC1 was reproducibly elevated in patients with unstable carotid plaques and showed robust discriminatory performance across independent cohorts. By integrating urinary proteomics with human plaque transcriptomic, single-cell, and tissue-level analyses, we further provided biological context for this association. Human plaque validation and macrophage assays further placed ADAMDEC1 within the biological context of vulnerable plaques and supported its association with macrophage-associated inflammatory features.

A major strength of this study lies in its stepwise design. We first established disease-associated urinary proteomic signatures in an unbiased discovery cohort, followed by internal validation and prioritization of reproducible candidates. These findings were subsequently confirmed in a large independent clinical cohort, in which urinary ADAMDEC1 demonstrated robust discriminatory capacity for unstable plaques. Importantly, this association remained evident after adjustment for conventional cardiovascular risk factors, supporting the independent association between urinary ADAMDEC1 and plaque vulnerability.

Previous efforts to identify biomarkers of carotid plaque instability have largely focused on circulating inflammatory mediators, proteases, and lipid-associated factors, including C-reactive protein, matrix metalloproteinases, and selected cytokines or chemokines (32, 33, 34). While several blood-based markers have been associated with plaque vulnerability, their clinical utility has been limited by modest specificity, susceptibility to systemic confounders, and variability across cohorts (5, 8, 32, 35). More recently, transcriptomic and proteomic analyses of excised carotid plaques have provided important insights into molecular features of vulnerable lesions (10, 36), yet these approaches are inherently invasive and unsuitable for longitudinal monitoring. In this context, our findings extend prior work by showing that urinary proteomic profiling can capture disease-relevant signatures associated with plaque instability, offering a noninvasive and repeatable approach for clinical assessment.

Importantly, the urinary proteomic changes were not limited to ADAMDEC1. Instead, they reflected coordinated biological processes that are well recognized in vulnerable plaque progression. Enrichment of extracellular matrix organization and collagen-containing matrix components is consistent with the extensive matrix remodeling that accompanies plaque destabilization. At the same time, pathways related to innate immunity, neutrophil degranulation, antigen processing and presentation, and major histocompatibility complex protein complexes point to broad immune activation associated with unstable lesions. Alterations in endocytosis- and protein-processing pathways further suggest disturbances in vesicular trafficking and proteostasis during plaque progression. Taken together, these findings suggest that the urinary proteome captures a systems-level molecular signature associated with plaque inflammation and tissue remodeling rather than reflecting a single biological pathway.

Within this broader proteomic landscape, ADAMDEC1 emerged as one of the most consistently prioritized proteins. Single-cell transcriptomic analysis localized *ADAMDEC1* expression predominantly to inflammatory macrophage subsets enriched in unstable plaques. This observation was further supported by immunohistochemical analyses, which demonstrated increased ADAMDEC1 protein abundance in macrophage-rich inflammatory regions. Together, these findings place urinary ADAMDEC1 within a biologically relevant context and support its association with macrophage-related inflammatory features of plaque instability.

ADAMDEC1 is a secreted glycosylated metalloproteinase of the atypical ADAM-like metzincin family that retains proteolytic activity (37). Although its physiological substrates and precise biological functions remain incompletely defined, available evidence links ADAMDEC1 more closely to inflammatory and immune-cell programs than to constitutive structural remodeling. Differential regulation of ADAMDEC1 has been reported across inflammatory and neoplastic conditions, supporting a broader role in immune regulation and tissue inflammatory responses (38, 39, 40, 41, 42). In the vascular field, elevated ADAMDEC1 expression was identified in ruptured carotid plaques as early as 2006 (43), and subsequent studies reported increased circulating ADAMDEC1 levels in patients with atherosclerosis, where elevated ADAMDEC1 has been reported to be associated with vascular smooth muscle cell proliferation, migration, and inflammatory activity (38). Recent work has further shown ADAMDEC1 expression in mature dendritic cells and macrophages (37), consistent with our single-cell analyses showing that *ADAMDEC1* expression is largely restricted to plaque macrophages and enriched in inflammatory macrophage subsets. Given the central role of macrophages in plaque initiation, progression, necrotic core expansion, rupture, and repair (44), these observations provide biological context for our findings. Protein-level validation in human plaque specimens further demonstrated higher ADAMDEC1 abundance in AC than in PA tissues, with IF showing colocalization with CD68-and CD86-positive macrophages. In THP-1–derived macrophages, rADAMDEC1 increased the proportion of M1-like cells, whereas ADAMDEC1 knockdown attenuated LPS-induced M1 polarization. Together, these results support an association between ADAMDEC1 and pro-inflammatory macrophage phenotypes in carotid atherosclerosis, although the downstream molecular mechanism remains unresolved.

Taken together, our findings support the translational potential of urinary ADAMDEC1 as a noninvasive biomarker of carotid plaque instability, but they should not be interpreted as evidence for immediate clinical implementation. Future prospective multicenter studies will be required to establish assay reproducibility, define pre-specified cutoff values, evaluate calibration and longitudinal predictive performance, and determine whether urinary ADAMDEC1 provides incremental value beyond established clinical and imaging-based risk stratification.

Several limitations should be acknowledged. The present study was cross-sectional, so we were unable to assess temporal changes in urinary ADAMDEC1 or determine whether it predicts future cerebrovascular events. Plaque classification relied on gross morphology together with H&E histology and independent pathological review. Although this provided a histologically grounded framework for distinguishing stable from unstable lesions, it is not an absolute gold standard and may not fully capture the spectrum of plaque vulnerability. In addition, paired plasma and plaque protein measurements were not available, limiting direct inference regarding the relationship between tissue, circulation, and urinary ADAMDEC1. Finally, while the current findings support a link between ADAMDEC1 and pro-inflammatory macrophage phenotypes, they do not establish the downstream molecular mechanism. Further longitudinal, cell-specific, and *in vivo* studies are therefore needed to clarify whether and how ADAMDEC1 participates in plaque inflammation and instability.

In summary, this study identifies urinary ADAMDEC1 as a reproducible, noninvasive biomarker associated with carotid plaque instability. By integrating urinary proteomics with human plaque transcriptomic, single-cell, tissue-level, and macrophage assay data, we provide an evidence framework linking urinary ADAMDEC1 to macrophage-associated inflammatory features in vulnerable plaques. These findings provide a rationale for evaluating urinary ADAMDEC1 as a complement to existing clinical and plaque-based assessments and establish a foundation for future longitudinal and mechanistic investigations.

## Ethics Approval and Consent to Participate

The study protocol was reviewed and approved by the Ethics Committee of the First Hospital of Jilin University (Approval No. 2025335). Written informed consent was obtained from all participants prior to their inclusion in the study.

## Consent for Publication

Not applicable.

## Data Availability

The mass spectrometry proteomics data generated in this study have been deposited to the iProX Consortium (ProteomeXchange) with the dataset identifier PXD064282 (https://proteomecentral.proteomexchange.org/cgi/GetDataset?ID=PXD064282). The public transcriptomic datasets analyzed during the current study are available in the Gene Expression Omnibus (GEO) repository under accession numbers GSE43292, GSE28829, and GSE159677. Any additional data supporting the findings of this study are available from the corresponding author upon reasonable request.

## Supplemental data

This article contains supplemental data.

## Conflict of interest

The authors declare no conflict of interest.
