# Supplementary material for: Urinary Proteomics Identifies ADAMDEC1 as a Non-invasive Biomarker of Unstable Carotid Atherosclerotic Plaques
Source: Mol Cell Proteomics. 2026 Jul 17;25(8):101624. doi: 10.1016/j.mcpro.2026.101624 (PMC13486800; doi:10.1016/j.mcpro.2026.101624)
Supplement: Supplementary file 1 [file mmc4.docx]

**Table of Contents**

**Supplementary Figure S1:** Representative macroscopic and histological characterisation of carotid atherosclerotic plaque phenotypes.

**Supplementary Table S1:** Variable DIA isolation window scheme used for data acquisition.

**Supplementary Table S2:** Complete protein identification and quantitative results for the discovery cohort.

**Supplementary Table S3:** Differentially expressed proteins identified in the discovery and validationcohorts.

**Supplementary Table S4:** Proteomic and transcriptomic studies of carotid atherosclerotic plaques in humans.

**Supplementary Table S5:** Demographic and clinical characteristics of the validation cohort stratified by plaque instability.

**Supplementary Table S6:** Multivariable logistic regression analysis of urinary ADAMDEC1 levels for predicting carotid plaque instability in the validation cohort.

**Supplementary Table S7:** Association of urinary ADAMDEC1 levels with conventional cardiovascular risk factors.

**Supplementary Fig. S1: Representative macroscopic and histological characterisation of carotid atherosclerotic plaque phenotypes.**

Left panels display the gross morphology of surgical specimens obtained from carotid endarterectomy (CEA). Right panels show the corresponding histological architecture via haematoxylin and eosin (H&E) staining. Top row: A representative stable plaque exhibiting a thick, intact fibrous cap with a smooth texture and a small lipid core. Bottom row: A representative unstable plaque characterised by a large necrotic core, evident intraplaque haemorrhage (reddish-brown areas in gross view), and surface ulceration. Plaque classification was independently verified by two pathologists based on modified AHA criteria. Scale bar = 1000 μm.

**Supplementary Table S1: Variable DIA isolation window scheme used for data acquisition.**

This table contains the complete DIA acquisition window scheme used for mass spectrometry analysis, including the precursor m/z values and corresponding isolation window widths for each variable window. Adjacent isolation windows were designed with a 1 m/z overlap, resulting in a total cycle time of 3.15 s. This table is provided as a separate Excel file due to its size.

**Supplementary Table S2: Complete protein identification and quantitative results for the discovery cohort.**

This table contains the complete list of proteins identified and quantified in the discovery cohort, including protein accession numbers, protein descriptions, gene symbols, the number of unique peptides assigned to each protein, and quantitative values for all samples. Only proteins identified with at least two unique peptides were retained for downstream quantitative analyses. This table is provided as a separate Excel file due to its size.

**Supplementary Table S3: Differentially expressed proteins identified in the discovery and validation cohorts.**

This table contains the full list of differentially expressed proteins (DEPs) identified in the training set (Sheet 1), the internal validation set (Sheet 2), and the common DEPs overlapping between both datasets (Sheet 3). Data include protein accession numbers, gene symbols, fold changes, log2 fold changes, and adjusted p-values. (This table is provided as a separate Excel file due to its size.)

**Supplementary Table S4: Proteomic and Transcriptomic Studies of Carotid Atherosclerotic Plaques in Humans**

| **Study** | **Year** | **Methods** | **Groups** | **Threshold value** |
| --- | --- | --- | --- | --- |
| **Nehme A et al. (27)** | 2019 | LC-MS/MS | 6 stable vs. 6 unstable plaques | FoldChange > 1.5 and *p* value < 0.05 |
| **Bao MH et al. (29)** | 2021 | HPLC-MS/MS | 5 stable vs. 5 unstable plaques | FoldChange > 2.0 and *p* value < 0.05 |
| **Vaisar T et al. (23)** | 2020 | LC-MS/MS | 6 stable vs. 5 unstable plaques | *p* value < 0.05 |
| **Lai Z et al. (21)** | 2024 | LC-MS/MS | 39 stable vs. 49 unstable plaques | FoldChange > 2.0 and *p* value < 0.05 |
| **Theofilatos K et al. (10)** | 2023 | LC-MS/MS | 69 symptomatic vs. 36 asymptomatic plaques | FoldChange > 2.0 and *p* value < 0.05 |
| **Lorentzen LG et al. (28)** | 2024 | LC-MS/MS | 7 symptomatic vs. 14 asymptomatic plaques | FoldChange > 1.0 and *p* value < 0.05 |
| **GSE28829** | 2011 | Array/GPL570 | 13 stable vs. 16 unstable plaques | N/A |
| **GSE43292** | 2013 | Array/GPL6244 | 32 macroscopically intact tissue vs. 32 atheroma plaques | N/A |

LC-MS/MS, liquid chromatography-tandem mass spectrometry; HPLC-MS/MS, tandem mass spectrometry hyphenated to liquid chromatography separation systems; FC, FoldChang.

**Supplementary Table S5: Demographic and clinical characteristics of the validation cohort stratified by plaque instability**

| **Characteristic** | **Validation cohort** | | ***p* value** |
| --- | --- | --- | --- |
|  | **Stable (n = 57)** | **Unstable (n= 83)** |  |
| **Age (years)** | 64 (59, 69) | 66 (61, 69) | 0.180 |
| **Sex, male, n (%)** | 42 (73.7) | 70 (84.3) | 0.122 |
| **BMI (kg/m2)** | 24.0 ± 2.9^a^ | 23.9 ± 2.6^a^ | 0.826 |
| **Smoking, n (%)** | 26 (45.6) | 45 (54.2) | 0.317 |
| **Drinking, n (%)** | 15 (26.3) | 33 (39.8) | 0.100 |
| **Hypertension, n (%)** | 37 (64.9) | 46 (55.4) | 0.261 |
| **Diabetes, n (%)** | 19 (33.3) | 14 (16.9) | 0.024 |
| **Coronary heart disease, n (%)** | 14 (24.6) | 19 (22.9) | 0.819 |
| **History of stroke, n (%)** | 10 (17.5) | 15 (18.1) | 0.936 |
| **Hyperlipidemia, n (%)** | 13 (22.8) | 21 (25.3) | 0.735 |
| **TG (mmol/L)** | 1.3 (1.0, 1.7) | 1.2 (0.9, 1.7) | 0.347 |
| **HDL (mmol/L)** | 1.0 (0.9, 1.1) | 1.0 (0.8, 1.1) | 0.971 |
| **LDL (mmol/L)** | 2.2 (1.6, 2.8) | 2.0 (1.8, 2.5) | 0.573 |
| **TC (mmol/L)** | 3.7 (2.7, 4.4) | 3.4 (3.1, 4.2) | 0.523 |

**Note:** Data are presented as median (IQR) for continuous variables and n(%) for categorical variables, unless otherwise indicated. ^a^Data for BMI are presented as mean ±SD. *p* values were calculated using the Mann–Whitney U test (or Student's t-test for BMI) for continuous variables and the Chi-square test for categorical variables.

**Abbreviations:** BMI, body mass index; HDL, high-density lipoprotein; LDL, low-density lipoprotein; TC, total cholesterol; TG, triglycerides.

**Supplementary Table S6: Multivariable logistic regression analysis of urinary ADAMDEC1 levels for predicting carotid plaque instability in the validation cohort**

| **Characteristic** | **Model 1** | | | **Model 2** | | | **Model 3** | | |
| --- | --- | --- | --- | --- | --- | --- | --- | --- | --- |
|  | **OR** | **95% CI** | ***p* value** | **OR** | **95% CI** | ***p* value** | **OR** | **95% CI** | ***p* value** |
| **ADAMDEC1/Cr (ng/mmol)** | 1.06 | 1.04, 1.09 | <0.001 | 1.06 | 1.04, 1.09 | <0.001 | 1.07 | 1.05, 1.10 | <0.001 |
| **Note:** Model 1: Unadjusted. Model 2: Adjusted for Age(years) and Sex. Model 3: Adjusted for Age(years), Sex, BMI(kg/m^2^), Smoking, Hypertension, Diabetes, and Hyperlipidemia. | | | | | | | | | |

**Abbreviations:** OR = Odds Ratio, CI = Confidence Interval

**Supplementary Table S7: Association of urinary ADAMDEC1 levels with conventional cardiovascular risk factors**

| **Characteristic** | **Stratified by ADAMDEC1 tertiles** | | | ***p* value** |
| --- | --- | --- | --- | --- |
|  | **Tertile 1, n = 17** | **Tertile 2, n = 57** | **Tertile 3, n = 66** |  |
| **Age(years)** | 64 (59, 67) | 65 (61, 69) | 65 (61, 69) | 0.685 |
| **Sex, male, n (%)** | 14 (82.4) | 43 (75.4) | 55 (83.3) | 0.555 |
| **BMI (kg/m^2^)** | 24.8 ± 3.5^a^ | 23.7 ± 2.3^a^ | 23.9 ± 2.8^a^ | 0.319 |
| **Smoking, n (%)** | 7 (41.2) | 34 (59.7) | 30 (45.5) | 0.205 |
| **Drinking, n (%)** | 5 (29.4) | 22 (38.6) | 21 (31.8) | 0.661 |
| **Hypertension, n (%)** | 12 (70.6) | 33 (57.9) | 38 (57.6) | 0.599 |
| **Diabetes, n (%)** | 4 (23.5) | 19 (33.3) | 10 (15.2) | 0.055 |
| **Coronary heart disease, n (%)** | 5 (29.4) | 13 (22.8) | 15 (22.7) | 0.820 |
| **History of stroke, n (%)** | 1 (5.9) | 10 (17.5) | 14 (21.2) | 0.373 |
| **Hyperlipidemia, n (%)** | 4 (23.5) | 10 (17.5) | 20 (30.3) | 0.266 |
| **TG (mmol/L)** | 1.46 (1.18, 1.89) | 1.21 (0.90, 1.68) | 1.24 (0.85, 1.63) | 0.507 |
| **HDL (mmol/L)** | 0.98 (0.93, 1.06) | 0.96 (0.81, 1.08) | 0.98 (0.84, 1.15) | 0.779 |
| **LDL (mmol/L)** | 2.22 (1.71, 2.68) | 2.07 (1.77, 2.74) | 2.01 (1.72, 2.52) | 0.818 |
| **TC (mmol/L)** | 3.57 (2.90, 4.17) | 3.49 (2.91, 4.35) | 3.48 (3.07, 4.15) | 0.947 |
| **Note:** Data are presented as median (IQR) for continuous variables and n (%) for categorical variables, unless otherwise indicated. *^a^*Data for BMI are presented as mean ± SD. **Abbreviations:** BMI, body mass index; HDL, high-density lipoprotein; LDL, low-density lipoprotein; TC, total cholesterol; TG, triglycerides. | | | | |
|  | | | | |
